# Genome-Wide Characterization of Glutathione Peroxidase (GPX) Gene Family in Rapeseed (*Brassica napus* L.) Revealed Their Role in Multiple Abiotic Stress Response and Hormone Signaling

**DOI:** 10.3390/antiox10091481

**Published:** 2021-09-17

**Authors:** Wei Li, Xuemin Huai, Peitao Li, Ali Raza, Muhammad Salman Mubarik, Madiha Habib, Sajid Fiaz, Binbin Zhang, Jun Pan, Rao Sohail Ahmad Khan

**Affiliations:** 1 Beijing Goldenway Bio-Tech Co., Ltd., Chaoyang District, Beijing 100015, China; huaixuemin@jiabowen.com (X.H.); lipeitao@jiabowen.com (P.L.); zhangbinbin@jiabowen.com (B.Z.); panjun@jiabowen.com (J.P.); 2 Fujian Provincial Key Laboratory of Crop Molecular and Cell Biology, Oil Crops Research Institute, Center of Legume Crop Genetics and Systems Biology, College of Agriculture, Fujian Agriculture and Forestry University (FAFU), Fuzhou 350002, China; 3 Centre of Agricultural Biochemistry and Biotechnology (CABB), University of Agriculture, Faisalabad 38040, Pakistan; msmubarik@gmail.com (M.S.M.); madihahabib217@gmail.com (M.H.); 4 Department of Plant Breeding and Genetics, The University of Haripur, Haripur 22600, Pakistan; sfiaz@uoh.edu.pk

**Keywords:** antioxidant, abiotic stress, genomics, gene expression, gene ontology, hormones, miRNA, rapeseed breeding

## Abstract

Plant glutathione peroxidases (GPXs) are the main enzymes in the antioxidant defense system that sustain H_2_O_2_ homeostasis and normalize plant reaction to abiotic stress conditions. To understand the major roles of the *GPX* gene family in rapeseed (*Brassica napus* L.), for the first time, a genome-wide study identified 25 *BnGPX* genes in the rapeseed genome. The phylogenetic analysis discovered that *GPX* genes were grouped into four major groups (Group I–Group IV) from rapeseed and three closely interrelated plant species. The universal investigation uncovered that the *BnGPXs* gene experienced segmental duplications and positive selection pressure. Gene structure and motifs examination recommended that most of the *BnGPX* genes demonstrated a comparatively well-maintained exon-intron and motifs arrangement within the identical group. Likewise, we recognized five hormones-, four stress-, and numerous light-reactive *cis*-elements in the promoters of *BnGPXs*. Five putative bna-miRNAs from two families were also prophesied, targeting six *BnGPXs* genes. Gene ontology annotation results proved the main role of *BnGPXs* in antioxidant defense systems, ROS, and response to stress stimulus. Several *BnGPXs* genes revealed boosted expression profiles in many developmental tissues/organs, i.e., root, seed, leaf, stem, flower, and silique. The qRT-PCR based expression profiling exhibited that two genes (*BnGPX21* and *BnGPX23*) were suggestively up-regulated against different hormones (ABA, IAA, and MeJA) and abiotic stress (salinity, cold, waterlogging, and drought) treatments. In short, our discoveries provide a basis for additional functional studies on the *BnGPX* genes in future rapeseed breeding programs.

## 1. Introduction

Plants are frequently exposed to several environmental stresses, comprising both abiotic and biotic, which significantly affect the growth and developmental processes [1,2,3,4]. These traumas can boost the production of reactive oxygen species (ROS), impairing cellular apparatuses and macromolecules, including DNA, proteins, and lipids, and eventually program the cells’ death [5,6,7]. In plants, ROS are chemical species formed by an inadequate drop of oxygen; these species are well thought out as key signaling molecules, regulating stress responses and contributing to plant production [6]. Primarily, ROS are produced in the apoplast, mitochondria, plasma membrane, chloroplast, peroxisomes, endoplasmic reticulum, and cell walls [5,6]. Consequently, plants have advanced enzymatic and nonenzymatic antioxidative defense systems to cope with the harmfulness of ROS [5,6,8].

Among different antioxidant enzymes, glutathione peroxidase (GPX; EC 1.11.1.9) is an effective ROS scavenging enzyme that goes to the non-heme thiol peroxidase family and observes glutathione (GSH) and thioredoxin (Trx) as dipping substrata [9]. In comparison to mammals GPXs, plants GPXs have a partiality to Trx as a replacement for GSH as the dipping substratum [10,11]. Furthermore, plants’ GPXs comprise cysteine (Cys) in their functioning sites, whereas usually, mammals’ GPXs comprise selenocysteine (SeCys) residue as a substitute to Cys [9,11,12].

In the recent past, several investigations have shown that the increased/up-regulated activity/expression of GPX enzyme and *GPX* genes help plants to cope with various environmental stresses. For instance, increased GPX activity helps different plants to improves tolerance to oxidative stress in cucumber (*Cucumis sativus* L.), tobacco (*Nicotiana tabacum*), and rice (*Oryza sativa* L.) seedlings [13]; water-deficit stress in wheat (*Triticum aestivum* L.) [14]; salicylic acid-induced salinity stress in tomato (*Solanum lycopersicum* L.) [15]; brassinosteroids-induced low-temperature stress tolerance in tomato [16]; etc. Similarly, the increased/up-regulated expression levels of *GPX* genes increased the tolerance to water-deficit stress in wheat [14]; drought tolerance in *Hevea* clones [17]; lead-induced oxidative stress tolerance in wheat [18]; etc. However, in some cases, the stressed plants also decreased the expression levels of *GPX* genes; for example, several *GPX* genes were down-regulated in response to drought stress in sorghum (*Sorghum bicolor* L.) [19]. The down-regulated expression of *GPX* genes could participate in the activation of some downstream mechanisms.

Some functional studies showed, for example, that the silencing of mitochondrial *GPX1* gene in rice activated the damage of photosynthesis against salinity stress [20]. Knockdown of two *GPX* genes, including *OsGPX1* and *OsGPX3*, could cruelly disturb the steady growth and developmental processes in rice [21]. Overexpression of *Rhodiola crenulata RcGPX5* gene raises drought stress tolerance in *Salvia miltiorrhiza* plants [22]. In another investigation, the overexpression of the *Arabidopsis AtGPXL5* gene resulted in changed plant growth and redox status under salinity stress in *Arabidopsis* [23]. In a nutshell, these studies specified that plant *GPX*s genes could regulate plant developmental processes, stress responses, tolerance mechanisms. 

Rapeseed (*Brassica napus* L.) is the second most significant oilseed crop and holds a complex genome. Various abiotic stresses meaningfully restrict rapeseed production [24,25,26,27]. To our best knowledge, the *GPX* gene family has not been documented in rapeseed. Hence, for the first time, the current study executed a genome-wide analysis to recognize *GPX* genes in the rapeseed genome. To get further insight into *GPX* genes evolution in rapeseed, their phylogenetic relations, synteny investigation, gene structures, conserved motifs, *cis*-elements, miRNA predictions, and functional annotation were categorized. Furthermore, their expression profile in several tissues/organs and under several hormones and abiotic stress environments were broadly evaluated, which profoundly boosted our knowledge of the *GPX* genes in rapeseed.

## 2. Materials and Methods

### 2.1. Documentation of GPX Gene Family in Rapeseed

As defined in recent reports [28,29], two methods were employed to classify *GPX* genes in the *Brassica napus* genome, including BLASTP and the Hidden Markov Model (HMM) [28]. The sequence of *Brassica napus* genome was downloaded from the BnPIR database (http://cbi.hzau.edu.cn/bnapus/index.php, accessed on 1 July 2021) [30]. For BLASTP, we used eight *Arabidopsis thaliana GPXs* (Table S1) amino acid sequences as an enquiry by e-value set to 1e^−5^. The amino acid sequences of eight *AtGPXs* were retrieved from the TAIR *Arabidopsis* genome database (http://www.arabidopsis.org/, accessed on 1 July 2021) [31]. Moreover, a local software, HMMER 3.1 [32], was used for hunting the *GPX* genes with default constraints. Then, the HMM profile of the GPX domain (Pfam: PF00255) obtained as of the Pfam database (http://pfam.xfam.org/, accessed on 1 July 2021) was employed to detect *GPX* gene sequences. Finally, 25 *BnGPX* genes were recognized by merging the two methods in the rapeseed genome. Furthermore, we recognized *GPX* genes in diverse plant species, i.e., *Brassica rapa* and *Brassica oleracea*, and their genome sequences downloaded from the JGI Phytozome 12.0 database (https://phytozome.jgi.doe.gov/pz/portal.html, accessed on 1 July 2021) [33] through a similar method.

### 2.2. Characterization of GPX Gene Family

The physico-chemical characteristics, including molecular weight and isoelectric points, were examined by means of the online ProtParam tool (http://web.expasy.org/protparam/, accessed on 1 July 2021) [34]. The subcellular localization of BnGPX proteins was prophesied from the WoLF PSORT server (https://wolfpsort.hgc.jp/, accessed on 1 July 2021) [35]. The genes structures of *BnGPX* was created via TBtools software (V 1.068; https://github.com/CJ-Chen/TBtools, accessed on 1 July 2021) [36]. The conserved motifs of BnGPX protein sequences were recognized by employing the MEME website (https://meme-suite.org/meme/db/motifs, accessed on 1 July 2021) [37].

### 2.3. Phylogenetic Tree and Synteny Analysis of BnGPX Family Proteins

To perceive the evolutionary relationship of the *BnGPX* gene family, we created a phylogenetic tree for *Brassica napus*, *Brassica oleracea*, *Brassica rapa*, and *A. thaliana* protein sequences. The sequence alignment was carried out using MEGA 7 software (https://megasoftware.net/home, accessed on 1 July 2021) [38]. The neighbor-joining (NJ) scheme was executed to develop a phylogenetic tree with 1000 bootstrap replicates using Evolview v3 website (https://www.evolgenius.info/evolview, accessed on 1 July 2021) [39] to show the phylogenetic tree. Synteny relationships of *GPX* genes were established by python-package, JCVI (https://github.com/tanghaibao/jcvi, accessed on 1 July 2021) [40] from *Brassica napus*, *Brassica oleracea*, *Brassica rapa*, and *Arabidopsis thaliana*. We precalculated the Ka/Ks ratios of all *GPXs* by employing KaKs_Calculator 2.0 software (https://sourceforge.net/projects/kakscalculator2/, accessed on 1 July 2021) [41].

### 2.4. Cis-Elements Analysis in the BnGPX Gene Promoters

To study the putative *cis*-elements in the *BnGPXs* promoters, we obtained the 2Kb sequence upstream of start codons in the *Brassica napus* genome. Then, the promoter sequence of the individual gene was examined using the PlantCARE website (http://bioinformatics.psb.ugent.be/webtools/plantcare/html/, accessed on 1 July 2021) [42], and the figure was drawn using TBtools (V 1.068) [36].

### 2.5. Estimation of Presumed miRNA Targeting BnGPX Genes and Functional Annotation Study

The coding sequence (CDS) of *BnGPXs* was used to recognize possible target miRNAs in the psRNATarget database (http://plantgrn.noble.org/psRNATarget/, accessed on 1 July 2021) [43] with default constraints. The interaction network between the miRNAs and *BnGPX* genes was drawn by Cytoscape software (V3.8.2) (https://cytoscape.org/download.html, accessed on 1 July 2021). Gene ontology (GO) annotation examination was accomplished by uploading all BnGPXs protein sequences to the eggNOG website (http://eggnog-mapper.embl.de/, accessed on 1 July 2021) [44]. Then, TBtools was used to execute the GO enrichment examination.

### 2.6. Expression Analysis of BnGPX Genes in Diverse Tissues

For tissue-specific expression profiling, we downloaded RNA-seq data (BioProject ID: PRJCA001495, accessed on 1 July 2021) of rapeseed from the National Genomics Data Center. The complete method was described in a recent work [28]. Mainly, cuffquant and Cuffnorm were employed to create normalized counts in transcripts per million (TPM) values. Based on TPM estimates, the expression heat map was formed using GraphPad Prism 8 software (https://www.graphpad.com/, accessed on 1 July 2021) [45].

### 2.7. Plant Material and Stress Conditions

In the current study, a typical cultivated variety, ZS11, was used for stress treatments. The seeds of the ZS11 genotype were obtained from Wuhan Zhongyou Seed Technology Co., Ltd., Wuhan, China. The stress treatments were performed as designated in previous work [28]. Briefly, the vigorous seeds were carefully chosen and sterilized with 10% hypochlorous acid solution for 5 min. The seeds were grown on water-saturated filter paper in a chamber (25 °C day/night and 16 h/8 h light/dark cycle) until the radicle’s extent turned up about 5 mm. For stress treatment, sprouted seeds were uncovered to 150 mM NaCl solution for salt stress, 15% PEG6000 solution for drought stress, and 4 °C for cold stress on water-saturated filter paper. For waterlogging stress, the seeds were flooded with water in the Eppendorf tube. To explore the influence of numerous hormones, the germinated seeds were planted in Murashige and Skoog (MS) medium supplied with 100 μM of each hormone such as abscisic acid (ABA), gibberellic acid (GA), methyl jasmonate (MeJA), and indole-acetic acid/auxin (IAA). The samples were collected at 0 (CK), 2, 4, 6, and 8 h after the treatments. Three biological repetitions were carried out for all the treatments. All the samples were rapidly frozen in liquid nitrogen and were kept at −80 °C for expression analysis.

### 2.8. RNA Extraction and qRT-PCR Analysis

Total RNA extraction and cDNA synthesis was performed using TransZol Up Plus RNA Kit (TransGen Biotech, Beijing, China) and cDNA Synthesis SuperMix (TransGen Biotech, Beijing, China) according to manufacturer instructions. The qRT-PCR was conducted with an ABI StepOne real-time fluorescence quantitative PCR instrument (Applied Biosystems, Foster City, CA, USA) using SYBR^®^ Green Supermix (Bio-Rad). The *BnACTIN* was used as an internal control. The qRT-PCR reaction was performed as follows: 94 °C for 10 min, followed by 40 cycles of 94 °C for 15 s, 60 °C for 30 s, and 72 °C for 10 s. Each qRT-PCR reaction was carried out with three biological triplicates, and the data were observed using the 2^−ΔΔCT^ method [28,46]. All the primers used in this experiment are listed in Table S1. The heatmap was formed using GraphPad Prism 8 software [45]. 

## 3. Results

### 3.1. Documentation and Characterization of GPX Gene Family in Rapeseed

Based on BLASTP examinations against the rapeseed genome employing eight Arabidopsis AtGPXs proteins as enquiries, a total of 25 presumed *GPX* genes were recognized in the complete rapeseed genome (Table 1). Henceforth, these genes are named “*BnGPX1-BnGPX25*”, among which 13 genes (*BnGPX1–BnGPX13*) were located on the A subgenome, and 12 genes (*BnGPX14*–*BnGPX25*) were located on the C subgenome (Table 1). Comprehensive data of the 25 *BnGPX* genes is shown in Table 1. In brief, gene length varied from 1028 bp (*BnGPX17*) to 4654 bp (*BnGPX9*) with 5–6 exons in each sequence (Table 1). Eleven genes comprise six exons, and 14 genes consist of five exons (Table 1). The CDS length varied from 363 bp (*BnGPX10*) to 726 bp (*BnGPX23*), whereas the protein length altered from 120 (BnGPX10) to 241 (BnGPX23) amino acids (Table 1). The predicted molecular weights of the 25 BnGPX proteins ranged from 13.45 kDa (BnGPX10) to 26.97 kDa (BnGPX22), and the isoelectric points ranged from 5.18 (BnGPX24) to 9.58 (BnGPX22) (Table 1). The subcellular localization results anticipated that 16 BnGPX proteins are located on the chloroplast, 6 proteins are located on the cytoplasm, 2 proteins (BnGPX3 and BnGPX9) are located on extracellular, and 1 protein (BnGPX20) is located on the nucleus (Table 1). Moreover, 8 *GPX* genes (*BolGPX1*–*BolGPX8*) from the *Brassica oleracea* and 12 *GPX* genes were also discovered from *Brassica rapa (BraGPX1*–*BraGPX12*) genomes (Table S2).

### 3.2. Phylogenetic Relationships of GPX Genes

**Figure 1:**
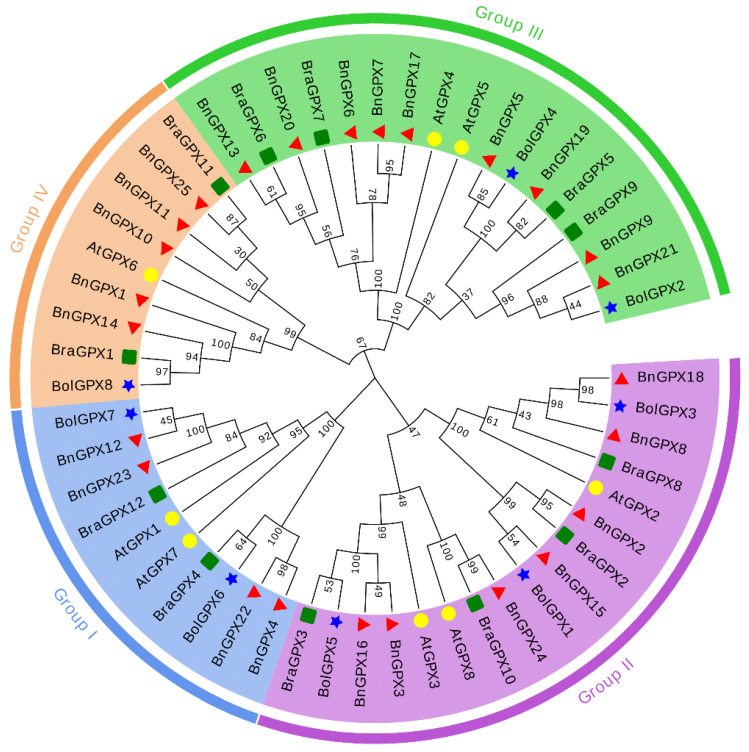
A neighbor-joining phylogenetic tree assessment of *GPX* genes in *Brassica napus*, *Brassica oleracea*, *Brassica rapa*, and *Arabidopsis thaliana.* Overall, 25 *BnGPXs* from *Brassica napus* (red triangle), 8 *BolGPXs* from *Brassica oleracea* (blue star), 12 *BraGPXs* from *Brassica rapa* (green box), and 8 *AtGPXs* from *Arabidopsis thaliana* (yellow circles) were clustered into four groups (Groups I–IV), symbolized by unique colors.

To discover the evolutionary history between the *BnGPX*, *BolGPX*, *BraGPX*, and *AtGPX* genes, an unrooted phylogenetic tree was assembled by a multiple sequence alignment (MSA) of the prophesied GPX protein sequences from *Brassica napus*, *Brassica oleracea*, *Brassica rapa*, and *Arabidopsis thaliana*, which was gathered into four main groups (Group I–Group IV) (Figure 1). The findings revealed that Group I was comprised of 10 *GPX* members (4 *BnGPXs*, 2 *BraGPXs*, 2 *BolGPXs*, and 2 *AtGPX*); Group II was comprised of 17 *GPX* members (7 *BnGPXs*, 4 *BraGPXs*, 3 *BolGPXs*, and 3 *AtGPX*); Group III contained 17 *GPX* members (9 *BnGPXs*, 2 *BolGPX*, 4 *BraGPXs*, and 2 *AtGPXs*); and Group IV was comprised of 9 *GPX* members (5 *BnGPXs*, 1 *BolGPXs*, 2 *BraGPXs*, and 1 *AtGPXs*) (Figure 1). Overall, *GPXs* clustering into the identical sub-group may possess parallel functions. It is worth mentioning that *BnGPX* genes were spread in each group with homologs from other plant species, and Group II and III were observed to have more *BnGPXs*, *BolGPXs*, *BraGPXs*, and *AtGPXs* members than the other three groups (Figure 1). Additionally, it was noticed that the *BnGPXs* have a stronger phylogenetic link with the *BolGPXs* and *BraGPXs* in each group. 

### 3.3. Evaluation of Chromosomal Dispersal and Synteny Links of BnGPX Genes

**Figure 2:**
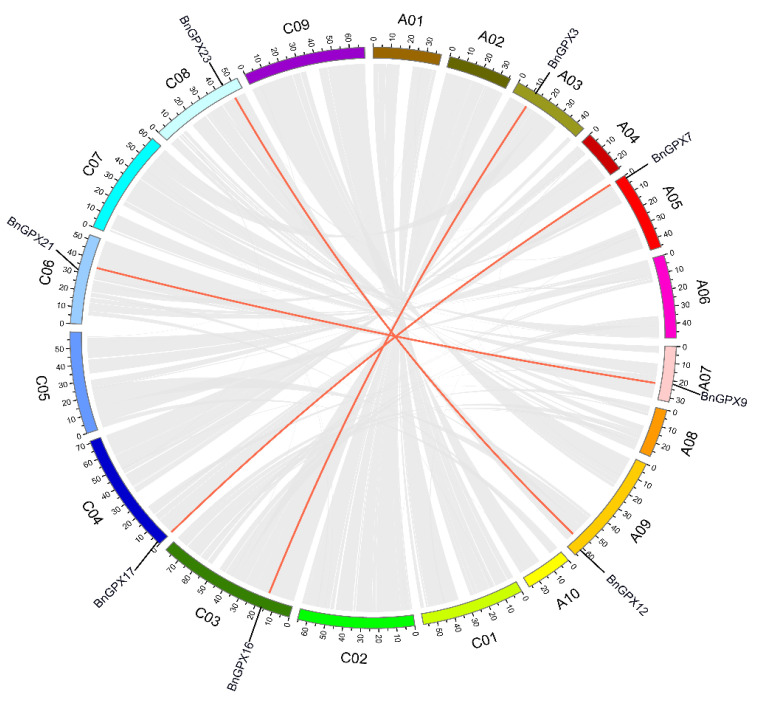
Circular drawings of the chromosomal distribution and inter-chromosomal organization of *BnGPX* genes. Gray lines in the background signify all the syntenic blocks in the rapeseed genome and the red lines indicate syntenic *GPX* gene pairs.

The development of new gene family followers in plants’ genome advancement is comparatively ascribed to tandem and segmental doubling events [47]. Thus, the corresponding events were explored in *BnGPXs* to clarify the rapeseed *GPX* gene duplication events. The chromosomal scatterings of 25 *BnGPX* genes were appraised. Notably, 8 out of the 19 chromosomes had *BnGPX* genes (Figure 2). Concisely, chromosomes A03, A05, A07, A09, C03, C04, C06, and C08 had only one *BnGPX* gene (Figure 2). Remarkably, the residual chromosomes did not have any *BnGPX* gene, and no tandem repeat paralogous genes were found in any regions. Moreover, eight paralogous genes were identified on the A03, A05, A07, A09, C03, C04, C06, and C08 chromosomes (Figure 2). These conclusions revealed that the duplication events played a critical role in developing the *BnGPX* family genes. 

**Figure 3:**
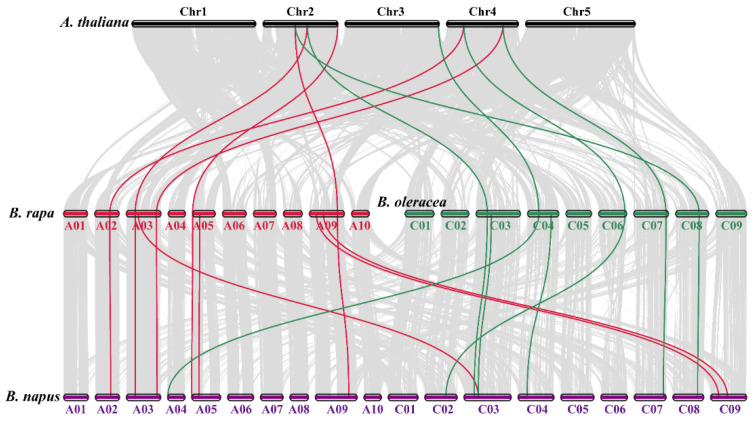
Synteny analysis of *GPX* genes in *Brassica napus*, *Brassica rapa*, *Brassica oleracea*, and *Arabidopsis thaliana* chromosomes. Gray lines in the background indicate the collinear blocks within *Brassica napus* and other plant genomes, whilst the red and green lines emphasize the syntenic *GPX* gene pairs. Genes located on the *Brassica napus* A and C sub-genomes are syntenic with *Brassica rapa*, *Brassica oleracea*, and *Arabidopsis thaliana*.

Collinearity analysis uncovered robust orthologs of the *GPX* genes among *Brassica napus* and the other hereditary plant species (Figure 3; Table S3). In short, in the A subgenome, 7 *Brassica napus* genes exhibited syntenic links with 5 *AtGPXs*, 1 *BraGPXs*, and *BolGPXs*. In the C subgenome, 9 *Brassica napus* genes presented syntenic links with 4 *AtGPXs*, 3 *BraGPXs*, and 2 *BolGPXs* (Figure 3). Particularly, numerous homologues of *Arabidopsis thaliana*, *Brassica rapa*, and *Brassica oleracea* withstood a syntenic alliance with *BnGPXs*, implying that whole-genome duplication (segmental duplication) events portrayed a vital role in *BnGPXs* gene family evolution in the rapeseed genome (Table S3).

The Ka/Ks ratio is a substantial index in estimating the replication events and selection pressures [48]. Thus, to comprehend the evolutionary constraints on the *BnGPX* gene family, the Ka, Ks, and Ka/Ks ratio was evaluated. The results stated that all the repeated *BnGPX* gene pairs had a Ka/Ks ratio of <1 (Table S3); however, two gene pairs (*BnGPX4/BraGPX4* and *BnGPX7/BraGPX7*) had a Ka/Ks ratio of >1 and experienced positive selection pressure (Table S3). Almost all genes experienced segmental duplication events. Similarly, *Brassica rapa*, *Brassica oleracea*, and *Arabidopsis thaliana* also experienced the purifying selective pressure (Table S3). Overall, the findings signify that the rapeseed *GPX* family genes might have encountered purifying and positive selection burden during the course of their evolution.

### 3.4. Evaluation of Gene Structures and Conserved Motifs of BnGPX Genes

**Figure 4:**
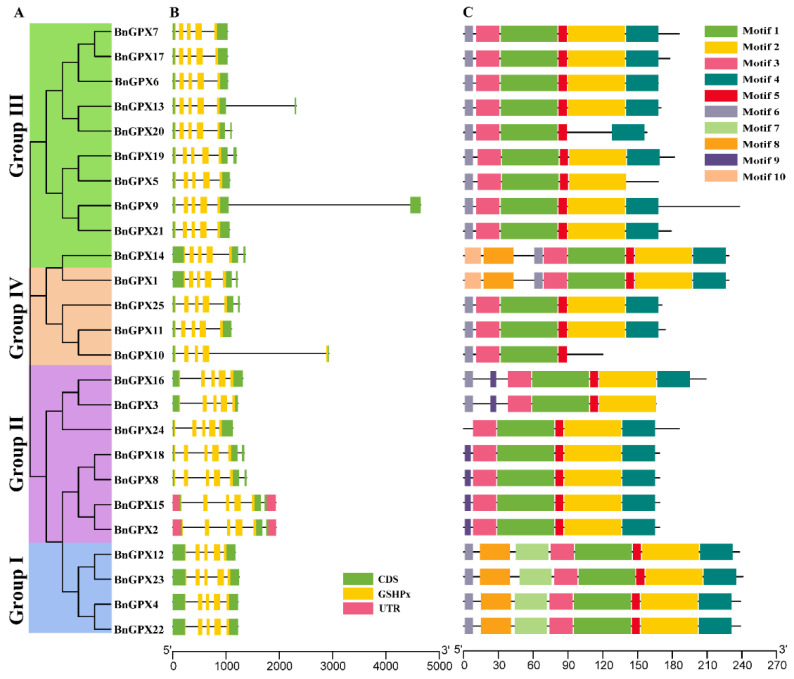
The gene structure and motif analysis of the *GPX* family genes from rapeseed. (**A**) Based on the phylogenetic interactions, the *BnGPX* genes are grouped into four groups (Groups I–IV). (**B**) The gene structure of the *BnGPXs*. Light green color indicates the CDS regions, pink color displays the UTR regions, yellow color displays the GSHPx domains, and black horizontal line demonstrates the introns. (**C**) Conserved motifs configurations are named in the *BnGPXs*. Unique color boxes demonstrate distinct motifs.

The gene structures (exon-intron arrangements) of the *BnGPX* genes were examined to gain insight into the expansion of rapeseed *GPX* family genes. The results showed that introns of the *BnGPXs* ranged from 4–5 (Figure 4A), and exons ranged from 5–6 in each sequence (Table 1). Group I contained 4 introns, whereas Groups II–IV include 4–5 introns. A similar trend was also observed for exons (5–6/gene) within the same groups (Figure 4A; Table 1). Mainly, *BnGPX2* and *BnGPX15* have diverse gene structures in Group II. In short, Groups II, III, and IV exhibited analogous intron-exon arrangements, and only Group I had a distinct intron-exon arrangement. These outcomes stipulated that *GPX* members within a group astonishingly had the same gene structures, steady with their phylogenetic relatives.

Additionally, we examined the full-length protein sequences of 25 *BnGPXs* to distinguish their conserved motifs. The conserved motif of the *BnGPX* genes varied from 4–8. Overall, ten conserved motifs were recognized, and the motif dispersals were also analogous within the groups (Groups I–IV) (Figure 4B; Table S4). For example, all members of Group I comprise 8; Group II contains 5–7; Group III includes 5–8; and Group IV encompasses 4–8 conserved motifs (Figure 4B). Notably, only *BnGPX10* had four motifs in Group IV (Figure 4B). Interestingly, motif 7 was specific to Group I; motif 9 was specific to Group II; motif 10 was limited to two genes (*BnGPX14* and *BnGPX1*) in Groups III and IV; and other motifs were nearly equally distributed on all the genes (Figure 4B). In a nutshell, the group organization’s evenness was strongly retained by exploring the conserved motifs dispersals, gene structures, and phylogenetic links, demonstrating that the GPX proteins have exceptionally preserved amino acid residues and members contained by a group might have analogous roles. 

### 3.5. Cis-Elements in the Promoters of BnGPX Genes

**Figure 5:**
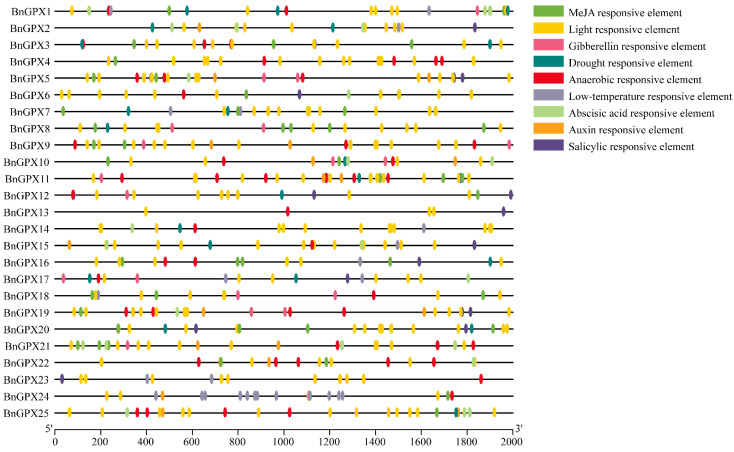
Evaluation of *cis*-regulatory elements in the *BnGPXs* promoters’ regions that are linked with various hormone- and stress-responsive elements. Unique color boxes display unique identified *cis*-elements.

To discriminate the gene functions and regulatory roles, *cis*-regulatory elements in *BnGPXs* promoter regions were inspected by searching a 2000 bp upstream region from each gene’s transcriptional activation site against the PlantCARE database. The complete data of *cis*-elements are offered in Table S5. The results display that five phytohormone-correlated (auxin, (abscisic acid (ABA), gibberellin (GA), methyl jasmonate (MeJA), and salicylic acid (SA)) responsive elements comprising TCA-element, TGA-element, CGTCA-motif, ABRE, AuxRR-core, TGACG-motif, TATC-box, GARE-motif, P-box, etc., were documented (Figure 5; Table S5). Predominantly, many phytohormone-connected elements were prophesied to be definite to some genes and extensively scattered (Figure 5), suggesting the decisive role of these genes in phytohormone arbitration.

Additionally, four abiotic stress-accompanying (drought, low-temperature, anaerobic, and light) elements, comprising ARE, LAMP-element, LTR, MRE, TCCC-motif, GT1-motif, chs-CMA1a, AE-box, box 4, G-box, MBS, etc. were recognized (Figure 5; Table S5). Primarily, several light-responsive elements were found to be extensively disseminated among all of the genes (Figure 5; Table S5), suggesting the extensive role of *BnGPXs* against light stress. On the whole, outcomes directed that *BnGPXs* expression levels may depart under phytohormone and abiotic stress environments.

### 3.6. Genome-Wide Investigation of miRNA Targeting BnGPX Genes

**Figure 6:**
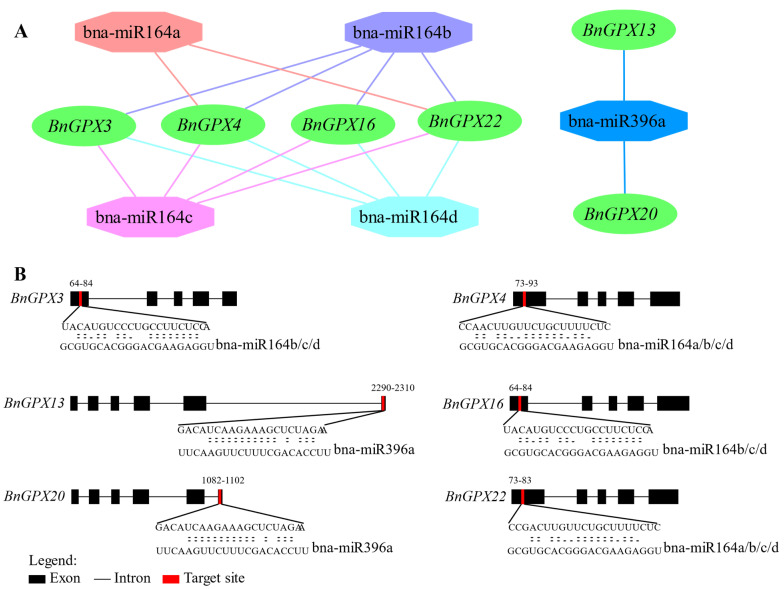
miRNA targeting *BnGPX* genes. (**A**) Network diagram of predicted miRNA targeting *BnGPX* genes. The green ellipse colors represent *BnGPXs* genes, and different color shapes signify miRNAs. (**B**) The graphic illustration signifies the *BnGPXs* gene targeted by miRNAs. The RNA sequence of each complementary site from 5′-3′ and the predicted miRNA sequence from 3′-5′ are visible in the long-drawn-out areas. See Table S6 for the comprehensive data of all predicted miRNAs.

Earlier reports demonstrated that miRNA-mediated regulation is associated with the stress responses in plants. Hence, to enhance our familiarity of miRNA-mediated post-transcriptional regulation on *BnGPXs*, the current study discovered five miRNAs targeting six *BnGPX* genes (Figure 6A; Table S6). The miRNA-targeted sites are shown in Figure 6B, and the comprehensive data of all miRNAs targeted sites/genes are introduced in Table S6. The outcomes disclosed that 4 members of the bna-miR164 family targeted four genes (*BnGPX3*, *BnGPX4*, *BnGPX16*, and *BnGPX22*); and one member of the bna-miR396 family targeted two genes (*BnGPX13* and *BnGPX20*) (Figure 6; Table S6). Primarily, *BnGPX3*, *BnGPX4*, *BnGPX16*, and *BnGPX22* were projected to be targeted by a superior number of miRNAs (Figure 6A; Table S6). The expression profiling of predicted miRNAs and their targeted sites/genes needs confirmation in the further investigation to oversee their biological functions in the rapeseed genome. 

### 3.7. Functional Annotation Evaluation of BnGPX Genes

To further distinguish the *BnGPX* genes’ roles, GO annotation and enrichment analysis were carried out based on biological process (BP), molecular function (MF), and cellular component (CC) classes. The BP, MF, and CC annotation conclusions presented abundant suggestively enriched terms (Table S7). For instance, the BP enrichment analysis uncovered that these genes were principally involved in response to hydrogen peroxide (GO:0042542), response to an organic substance (GO:0010033), cellular response to abiotic stimulus (GO:0071214), response to reactive oxygen species (GO:0000302), etc. (Table S7). Results of MF annotation revealed that these genes are involved in oxidoreductase activity (GO:0016684), antioxidant activity (GO:0016209), molecular_function (GO:0003674), glutathione peroxidase activity (GO:0004602), peroxidase activity (GO:0004601), etc. (Table S7). These terms also validate the function of *BnGPX* genes in ROS scavenging and antioxidant defense systems. The CC enrichment analysis confirms that these genes are mainly linked with cellular anatomical entity (GO:0110165), cytoplasm (GO:0005737), obsolete intracellular part (GO:0044424), obsolete cell part (GO:0044464), and organelle (GO:0043226), etc. (Table S7). Notably, few of these terms follow the prediction of subcellular localization of the GPX proteins. In short, CC, BP, and MF-GO annotation results proved the main role of *BnGPXs* in antioxidant defense systems, ROS, and response to stress stimulus.

### 3.8. Tissue-Specific Expression Profiles of BnGPX Genes in Rapeseed

**Figure 7:**
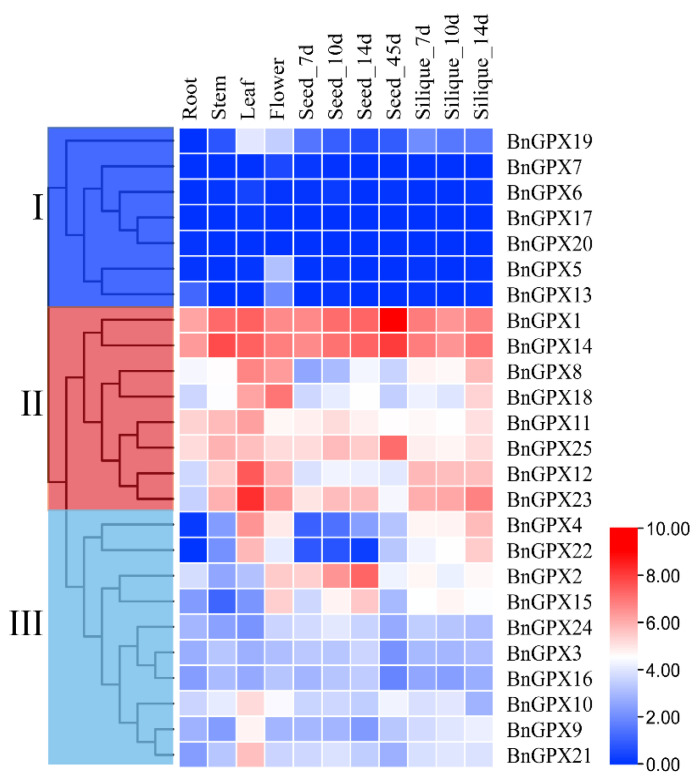
Expression profiles of *BnGPX* genes in numerous tissues at different growth stages of rapeseed. The 7d, 10d, 14d, and 45d tags indicated the time-points when the samples were harvested. The red color indicates high and the blue color indicates low expression levels. The expression heat map was generated based on transcripts per million (TPM) values.

The tissue-specific expression profiles of *BnGPXs* genes were observed in six diverse tissues and organs, i.e., roots, stems, leaves, flowers, seeds, and silique, using RNA-seq data from *Brassica napus* (ZhongShuang 11 variety) (BioProject ID PRJCA001495). The expression profiles of the *BnGPX* genes altered in the different tissues and organs. As illustrated in Figure 7, group II genes exhibit higher expression in all of the tissues except *BnGPX8*, *BnGPX12*, and *BnGPX18*, which displayed relatively lower expression in seeds (Figure 7). On the other hand, group I and III genes show relatively lower expression in all of the tissues except *BnGPX2*, *BnGPX4*, *BnGPX15*, and *BnGPX22* that showed somehow higher expression in leaf, flower, seeds, and silique (Figure 7). Thus, it is noteworthy that group II genes may play substantial roles in rapeseed developmental processes. 

### 3.9. Response of BnGPX Genes to Different Hormones and Abiotic Stress Treatments

**Figure 8:**
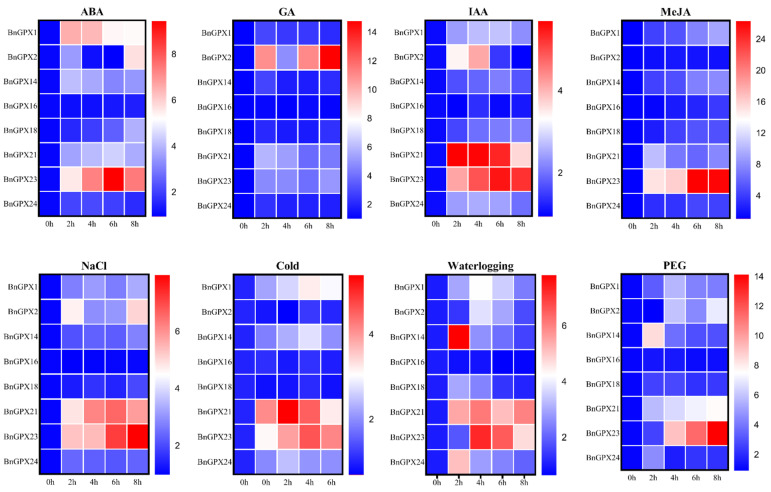
Expression profiles of *BnGPX* genes expression against various hormone and abiotic stress conditions at different time points (0 (CK), 2, 4, 6, and 8 h). The red color shows high and the blue color shows low expression levels. The expression bars present the comparative gene expressions based on the 2^−ΔΔCT^ method.

To investigate the *BnGPX* genes’ expression profiles under various hormones (ABA, GA, IAA, and MeJA) and abiotic stress (salinity, cold, waterlogging, and drought) conditions, the qRT-PCR-based expression profiling of 8 randomly chosen *BnGPX* genes was performed (Figure 8). In response to all of these stress environments, *BnGPX21* and *BnGPX23* were up-regulated under IAA (5.68, 5.64, 5.33, and 3.68 folds for *BnGPX21*; 4.13, 4.91, 5.49, and 5.22 folds for *BnGPX23*), salinity (4.83, 6.14, 6.54, and 5.81 folds for *BnGPX21*; 5.28, 5.40, 7.11, and 7.96 folds for *BnGPX23*), cold (4.09, 5.38, 4.5, and 3.21 folds for *BnGPX21*; 3.1, 3.9, 4.6, and 4.14 folds for *BnGPX23*), waterlogging (5.44, 6.09, 5.14, and 5.97 folds for *BnGPX21*; 1.84, 7.24, 6.51, and 4.70 folds for *BnGPX23*), and drought (5.71, 6.51, 7.15, and 7.59 folds for *BnGPX21*; 2.63, 9.09, 11.37, 14.10 folds for *BnGPX23*) conditions at 2, 4, 6, and 8 h, respectively, compared to CK (Figure 8), whereas *BnGPX23* was also up-regulated under ABA (5.55, 7.27, 9.43, and 7.39 folds) and MeJA (14.97, 16.01, 26.26, and 26.03 folds) treatments at 2, 4, 6, and 8 h, respectively, compared to CK (Figure 8). Under GA treatment, only *BnGPX2* was up-regulated (10.9, 11.04, and 14.75 folds) at 2, 6, and 8 h, respectively, compared to CK. On the other hand, *BnGPX1* and *BnGPX2* showed considerable expression under ABA, IAA, salinity, waterlogging, and drought conditions at certain time points (Figure 8). In comparison, the rest of the genes showed lower expression under all of the stress conditions. The results signify that mainly *BnGPX21* and *BnGPX23* genes could play a vital role in alleviating the harmful impact of different hormones and abiotic stress conditions in rapeseed. 

## 4. Discussion

Rapeseed is an allotetraploid crop that practices widespread genome replication and integration activities [49]. However, rapeseed yield is influenced by numerous abiotic pressures, including temperature (cold/heat), high salinity, drought, waterlogging, and heavy metals toxicity [24,27,50]. The generation of ROS in response to normal and stress atmospheres is assessed and scavenged via GPXs, which is considered one of the most effective ROS antioxidant scavenging enzymes [6,9]. Further, GPX enzyme and *GPX* genes also contribute to several plant physiological, biochemical, and molecular mechanisms to withstand harsh environmental stresses [5,6]. In the recent past, *GPX* gene families have been reported in numerous crop plants, including five *GPX* genes in date palm (*Phoenix dactylifera* L.) [51] and in *Rhodiola crenulate* [52]; six genes in cucumber (*Cucumis sativus* L.) [53], watermelon (*Citrullus lanatus* L.) [54], and *Lotus japonicus* [55]; seven genes in sorghum (*Sorghum bicolor* L.) [19]; eight genes in *Thellungiella salsuginea* [56] and peach fruit (*Prunus persica* L.) [57]; 13 genes in cotton (*Gossypium hirsutum* L.) [58]; and 14 genes in bread wheat [59]. To the best of our knowledge, the *GPX* gene family was yet to be reported in rapeseed that responds to multiple stress conditions. The availability of the entire rapeseed genome justifies the genome-wide description of the *GPX* gene family, which could be used for forthcoming rapeseed breeding programs.

According to the available literature, the current study identified the largest *GPX* gene family (25 *BnGPXs*) in the rapeseed genome (Table 1), which were grouped into four major groups (Figure 1). Deviations in the *GPXs* gene numbers between different plant species may be attributed to gene replication experiences, involving tandem and segmental replications, and participate in spreading *GPXs* for deviation. Gene replication of *GPX* genes was also uncovered in different plant species [19,57,58]. The current study showed that *BnGPXs* underwent segmental duplication, purifying and positive selection burden during their evolution (Table S3). Subsequently, these consequences recommended that *BnGPXs* replication activities might play an imperative role in gene evolution.

The phylogenetic tree showed that *GPXs* genes from rapeseed and other three plant species, including *Brassica rapa*, *Brassica oleracea*, and *Arabidopsis thaliana*, were categorized into four main groups (Figure 1), which was constant with the grouping in other plant species such as *Thellungiella salsuginea* [56], cotton [58], sorghum [19], and bread wheat [59]. Fascinatingly, each group comprised one or more *GPXs* genes from all plant species, and every *BnGPX* was extremely correlated to its homologs in the other three plant species, particularly in *Brassica rapa* and *Brassica oleracea*, suggesting that the mutual ancestor was self-possessed of pairs of possible orthologs. Additionally, gene structure analysis discovered that most of the *BnGPX* genes owned five introns (Figure 4; Table 1); however, the number of exons ranged widely from four to five, and introns number varied from five to six in each group (Figure 4). Similar findings were also reported in cucumber, where all *GPX* genes had four to five introns and four to five exons [53]. In cotton, six exons and five introns were found in 13 *GPX* genes [58]. The exon-intron gatherings’ divergence was practiced by three essential methods (exon/intron-gain/loss, exonization/pseudoexonization, and insertion/deletion), and they are precisely supported to structural divergence [60]. Remarkably, the *GPX* genes in each group offered parallel exon-intron organization and conserved motifs (Figure 4), indicating that these genes may take part in the matching roles associated with multiple abiotic cues. Similar discoveries have also been described in *Thellungiella salsuginea* [56], cotton [58], sorghum [19], and bread wheat [59].

To better comprehend the role of the *BnGPX* genes in contradiction of some environmental factors, *cis*-elements in the promoter regions were predicted. The outcomes demonstrated that two major kinds of *cis*-elements were identified, i.e., stress- and phytohormone-responsive (Figure 5; Table S5). Almost all recognized *cis*-elements were allied with phytohormones (ABA, MeJA, GA, SA, and auxin), and abiotic stress (drought, low-temperature, light, and anaerobic induction) (Table S5). The previous report suggests that *cis*-elements support plant stress responses [61]. Moreover, these gene functions were additionally deep-rooted by the GO annotation investigation (Table S7). Our results are in agreement with previous reports, where *GPX* genes contributed to several stress responses [19,51,55,56,59]. These conclusions can increase our comprehension of *BnGPX* genes under varied environmental circumstances.

In recent years, ample miRNAs have been documented by genome-wide investigation in rapeseed to take part in various environmental issues [62,63,64,65,66]. None of the previously reported *GPX*s gene families in different plants has reported miRNA-targeting *GPX* genes. Therefore, for the first time, the current study discovered five miRNAs from two families (miR164 and miR396) targeting six *BnGPX* genes (Figure 6; Table S6). In a recent study, *Vm-milR37* subsidized in pathogenicity through regulating the *VmGPX* gene, which takes part in the oxidative stress response throughout *Valsa mali* infection [67]. Notably, there is no report on miRNAs targeting *GPX* genes in plants. However, the identified miRNAs have been documented in different plants and stress conditions. For instance, miR164 regulated the salinity stress tolerance mechanism in maize (*Zea mays* L.) [68]. In another study, miR164-targeted *NAC* genes undesirably regulated drought stress tolerance in rice plants [69]. Further, miR164 negatively regulated the resistance of wheat to stripe rust [70]. Similarly, another miRNA (miR396) arbitrated amendment in plant growth and salt stress responses in creeping bentgrass (*Agrostis stolonifera*) [71]. Further, miR396 was found to be elaborated in plant response to vernalization and flower advancement in *Agrostis stolonifera* [72]. In another study, the overexpression of osa-MIR396c declines salinity and alkali stress tolerance in rice [73]. It is worth mentioning that these findings support our outcomes and endorse that bna-miRNAs might play critical roles in the contradiction of various stresses by fluctuating the transcript profiles of *GPX* genes in rapeseed.

Growing evidence specifies that *GPXs* genes may display diverse expression profiles in different organs/tissues and under stress circumstances [19,51,55,56,59]. Hence, the tissue-specific expression profiles of *BnGPXs* genes were evaluated in six diverse developmental tissues using RNA-seq data (Figure 7), and these results are in agreement with previous reports. For example, six watermelon *ClGPX* genes presented higher expressions in flowers and fruits [54]. In cotton, almost all *GhGPXs* genes exhibited the uppermost expression in flowers [58]. In cucumber, some *CsGPX* genes showed higher expression in roots and flowers [53]. The innumerable expression profiles of *BnGPX* genes (mainly from Group II) specify that they may play diverse purposeful roles at certain developmental procedures in rapeseed.

Similarly, the expression profiles of eight *BnGPX* genes were appraised under diverse phytohormones and abiotic stress treatments (Figure 8). Among all evaluated genes, *BnGPX21* and *BnGPX23* showed higher expression under almost all stress treatments except GA (Figure 8). These consequences agreed with earlier discoveries where several *GPX* genes exhibited sophisticated expression against stress treatments. For example, in watermelon, almost all *ClGPX* genes were up-regulated in response to cold, drought, and salinity stresses [54]. Nearly all *T. salsuginea TsGPXs* genes were suggestively up-regulated at a one-time point under salinity and drought treatments [56]. *Pennisetum glaucum PgGPX* expression levels were highly induced by salinity and drought conditions [12]. In cucumber, almost all six *CsGPX* genes were significantly up-regulated at some time points against cold, salinity, drought, and ABA treatments [53]. These discoveries offered robust evidence that *GPX* genes play a well-maintained role in defending abiotic and hormone stresses in diverse plant species.

## 5. Conclusions

In conclusion, a total of 25 *BnGPX* genes were identified in the rapeseed genome. To gain in-depth insights into the evolution of the *GPX* gene family in rapeseed genome, gene structure, phylogenetic and synteny, conserved motifs, *cis*-elements, GO annotation, miRNA prediction, tissue-specific expression, and expression profiling against diverse hormones, and abiotic stress treatments were carried out. The *BnGPX* genes were differentially expressed in numerous tissues/organs, signifying that these genes might contribute precise roles in rapeseed development. The outcomes of gene expression profiling in response to diverse abiotic stresses and hormones treatments discovered that several *BnGPX* genes might contribute to stress responses and hormone signaling pathways. These discoveries will lay the basis for studying the roles of *BnGPX* genes in rapeseed developmental processes and response to several stresses using different functional validation options, such as overexpression, knockout via CRISPR/Cas system, etc. 

## Figures and Tables

**Table 1:** Information of the 25 *BnGPX* genes discovered in rapeseed genome.

Gene ID	Gene Name	Gene Position (bp)	Gene Length (bp)	CDS Length (bp)	Exon	Protein Length (AA)	Molecular Weight (kDa)	Isoelectric Point (pI)	Subcellular Localization
BnaA02T0266400ZS	*BnGPX1*	A02:18048650-18049870 −	1220	690	6	229	25.28	9.21	Chloroplast
BnaA03T0152400ZS	*BnGPX2*	A03:7777692-7779631 −	1939	510	6	169	18.92	6.34	Cytoplasm
BnaA03T0210900ZS	*BnGPX3*	A03:11008433-11009656 −	1223	501	5	166	18.39	8.92	Extracellular
BnaA03T0529000ZS	*BnGPX4*	A03:29805209-29806435 +	1226	720	5	239	26.84	9.46	Chloroplast
BnaA04T0003000ZS	*BnGPX5*	A04:177901-178974 −	1073	507	5	168	18.85	8.82	Chloroplast
BnaA04T0301400ZS	*BnGPX6*	A04:25656674-25657708 −	1034	507	5	168	18.99	8.99	Chloroplast
BnaA05T0000100ZS	*BnGPX7*	A05:5755-6787 −	1032	561	5	186	21.00	8.86	Cytoplasm
BnaA05T0124600ZS	*BnGPX8*	A05:7501774-7503157 +	1383	510	6	169	18.93	6.34	Cytoplasm
BnaA07T0222500ZS	*BnGPX9*	A07:22717658-22722312 +	4654	717	6	238	26.86	9.68	Extracellular
BnaA08T0269900ZS	*BnGPX10*	A08:25981327-25984256 −	2929	363	5	120	13.45	6.28	Chloroplast
BnaA09T0247900ZS	*BnGPX11*	A09:18151781-18152884 −	1103	525	5	174	19.09	6.81	Chloroplast
BnaA09T0577200ZS	*BnGPX12*	A09:58245269-58246443 −	1174	717	5	238	26.45	9.23	Chloroplast
BnaA09T0721600ZS	*BnGPX13*	A09:65850924-65853242 +	2318	513	6	170	19.20	9.17	Chloroplast
BnaC02T0362600ZS	*BnGPX14*	C02:37887177-37888540 −	1363	690	6	229	25.36	9.3	Chloroplast
BnaC03T0178400ZS	*BnGPX15*	C03:10008604-10010540 −	1936	510	6	169	18.99	5.6	Cytoplasm
BnaC03T0248900ZS	*BnGPX16*	C03:15285876-15287196 −	1320	630	5	209	23.29	8.76	Chloroplast
BnaC04T0001100ZS	*BnGPX17*	C04:225671-226699 +	1028	537	5	178	20.16	9.13	Cytoplasm
BnaC04T0156100ZS	*BnGPX18*	C04:14243676-14245017 +	1341	510	6	169	18.93	6.34	Cytoplasm
BnaC04T0257400ZS	*BnGPX19*	C04:36128046-36129247 −	1201	549	6	182	20.30	8.58	Chloroplast
BnaC04T0617900ZS	*BnGPX20*	C04:71165478-71166588 +	1110	477	6	158	18.10	9.05	Nucleus
BnaC06T0239400ZS	*BnGPX21*	C06:34932165-34933231 +	1066	540	5	179	20.22	9.18	Chloroplast
BnaC07T0506700ZS	*BnGPX22*	C07:58481304-58482530 +	1226	720	5	239	26.97	9.58	Chloroplast
BnaC08T0428600ZS	*BnGPX23*	C08:47326442-47327687 −	1245	726	5	241	26.71	9.33	Chloroplast
BnaC09T0141600ZS	*BnGPX24*	C09:10653827-10654957 +	1130	561	5	186	21.09	5.18	Chloroplast
BnaC09T0292200ZS	*BnGPX25*	C09:28600420-28601680 −	1260	516	6	171	18.80	8.32	Chloroplast

In the genomic position, the positive (+) and negative (−) signs indicate the existence of a gene on the positive and negative strand of that specific marker, respectively.

## Data Availability

The datasets used and/or analyzed during the current study are shown in the supplementary files. For tissue-specific expression profiling, we downloaded RNA-seq data of rapeseed (BioProject ID: PRJCA001495, accessed on 1 July 2021) from the National Genomics Data Center. The rapeseed genome sequence was downloaded from BnPIR database (http://cbi.hzau.edu.cn/bnapus/index.php, accessed on 1 July 2021). The sequences of *Brassica rapa* and *Brassica oleracea* are available at Phytozome database (https://phytozome.jgi.doe.gov/pz/portal.html, accessed on 1 July 2021).

## Supplementary Materials

The following are available online at https://www.mdpi.com/article/10.3390/antiox10091481/s1, Table S1: A list of primers used for gene expression analysis in qRT-PCR, Table S2: The sequence of *GPX* family genes in *Brassica napus*, *Arabidopsis thaliana*, *Brassica oleracea*, and *Brassica rapa*, Table S3: The data of gene duplication type, Ka, Ks, and Ka/Ks ratio values of *B. napus* and other three plant species, Table S4: Information on the 10 identified motifs in BnGPX proteins, Table S5: Information on hormone- and stress-related *cis*-elements identified in the promoters regions of *BnGPXs*, Table S6: Information on miRNA targeted *BnGPX* genes, Table S7: The GO enrichment analysis of *BnGPX* genes.
